# Extracting causal relations on HIV drug resistance from literature

**DOI:** 10.1186/1471-2105-11-101

**Published:** 2010-02-23

**Authors:** Quoc-Chinh Bui, Breanndán Ó Nualláin, Charles A Boucher, Peter MA Sloot

**Affiliations:** 1 Computational Science, University of Amsterdam, Science Park 107, 1098 XG Amsterdam, The Netherlands; 2 Department of Virology, Erasmus University Rotterdam, Dr Molewaterplein 50, 3015 GE, Rotterdam, The Netherlands

## Abstract

**Background:**

In HIV treatment it is critical to have up-to-date resistance data of applicable drugs since HIV has a very high rate of mutation. These data are made available through scientific publications and must be extracted manually by experts in order to be used by virologists and medical doctors. Therefore there is an urgent need for a tool that partially automates this process and is able to retrieve relations between drugs and virus mutations from literature.

**Results:**

In this work we present a novel method to extract and combine relationships between HIV drugs and mutations in viral genomes. Our extraction method is based on natural language processing (NLP) which produces grammatical relations and applies a set of rules to these relations. We applied our method to a relevant set of PubMed abstracts and obtained 2,434 extracted relations with an estimated performance of 84% for F-score. We then combined the extracted relations using logistic regression to generate resistance values for each <drug, mutation> pair. The results of this relation combination show more than 85% agreement with the Stanford HIVDB for the ten most frequently occurring mutations. The system is used in 5 hospitals from the Virolab project http://www.virolab.org to preselect the most relevant novel resistance data from literature and present those to virologists and medical doctors for further evaluation.

**Conclusions:**

The proposed relation extraction and combination method has a good performance on extracting HIV drug resistance data. It can be used in large-scale relation extraction experiments. The developed methods can also be applied to extract other type of relations such as gene-protein, gene-disease, and disease-mutation.

## Background

The Human immunodeficiency virus (HIV) is the cause of acquired immunodeficiency syndrome (AIDS). HIV infection is now recognized as a pandemic. As of January 2006 the World Health Organization estimate that AIDS has killed over 25 million people since it was first recognized in 1981 [1]. Treatment of HIV infection consists of highly active antiretroviral therapy (HAART), a multi-drug treatment and has been shown to be effective in suppressing viral replication in many patients. However, the long-term use of these drugs leads to drug resistance caused by the viral mutations that occur under drug pressure. The resulting treatment failure requires new treatment regimens that can suppress the new mutations [2]. Therefore, in HIV treatment, it is critical to have up-to-date drug resistance data for selecting a treatment regimen to which the virus is still susceptible in the presence of resistant mutations.

To assist physicians in selecting the most suitable treatment regimen, currently there are two methods available to predict HIV drug resistance: a rule-based approach [3] and recently a computational approach [4,5]. For the former systems such as Stanford HIVDB http://hivdb.stanford.edu and RegaDB http://www.rega.kuleuven.be/cev/regadb, HIV drug resistance data are updated with resistance data manually gleaned from scientific publications by experts in this field. However, the amount of biomedical literature regarding to HIV drug resistance is increasing rapidly and it is becoming highly labour intensive for experts to collect reliable drug resistance information in a convenient and effective manner. Thus, a significant amount of drug resistance data remains hidden in biomedical literature. Therefore there is a need for computational methods that automate parts of this process and that can assist in retrieving and updating causal relations between drugs and virus mutations from literature.

Several approaches for extracting relations of interest (e.g. protein-protein, gene-protein) in biomedical texts have been reported [6]. The approaches range from co-occurrence to natural language processing (NLP) techniques. Co-occurrence is the simplest approach for relation extraction of entities within sentences. It assumes that if two entities are repeatedly mentioned together, they are somehow related. This approach provides high sensitivity (measuring the 'coverage') but very low specificity (measuring the accuracy) [7].

Other approaches use pattern-based techniques to extract relations that increase specificity, unfortunately at the cost of significantly lower sensitivity [8]. The patterns are either manually defined or automatically learned through annotated data. Manual patterns are generated by domain experts through the analysis of entities connected by a specific relation from text. Automatic patterns are generated by learning from text surrounding entity pairs known to have the relationship of interest. However, the more detailed the analysis of the text, the more patterns must be taken into account to deal with the large amount of surface grammatical variation in the texts [9].

Systems that are based on NLP techniques use either shallow parsing, which divides the sentence into chunks [10,11] or full parsing, which provides complete syntactic analysis of sentence structures. Since full parsing produces more elaborate syntactic information than shallow parsing, relation extraction systems based on full parsing can potentially provide better results [12]. The output of the parser is represented as constituent parse trees or dependency parse trees. Based on syntactic patterns or the shortest path between entities in the dependency trees, two approaches can then be applied to extract relations from parse trees: either a rule set which is manually defined [13-16] or machine learning techniques (e.g. SVM) are used [17-19].

Recent relation extraction methods focus on extraction of protein-protein interactions or protein-gene interactions [20-22]; a limited number of methods also deal with contradiction of extracted relations by assigning a strength score based on the amount of contradiction [23,24]. Much less attention has been paid to the extraction of other types of relationships and combination of extracted relations: this research area still remains largely untouched [25].

In this paper, we introduce a novel method to extract and combine relationships between mutations in viral genomes and HIV drugs, hereafter referred to as causal relations, which express changes in the resistance to the HIV drugs which are attributed to the presence or absence of certain mutations on the HIV genome. Our system distinguishes itself from previous research on relation extraction in a number of ways. First, we apply rules to extract relations from grammatical relations of sentence constituents. Next, we combine extracted relations to generate a unique resistance value for each <drug, mutation> pair. To the best of our knowledge, this is the first attempt to apply automatic relation discovery in the field of HIV drug-ranking.

## Methods

**Figure 1:**
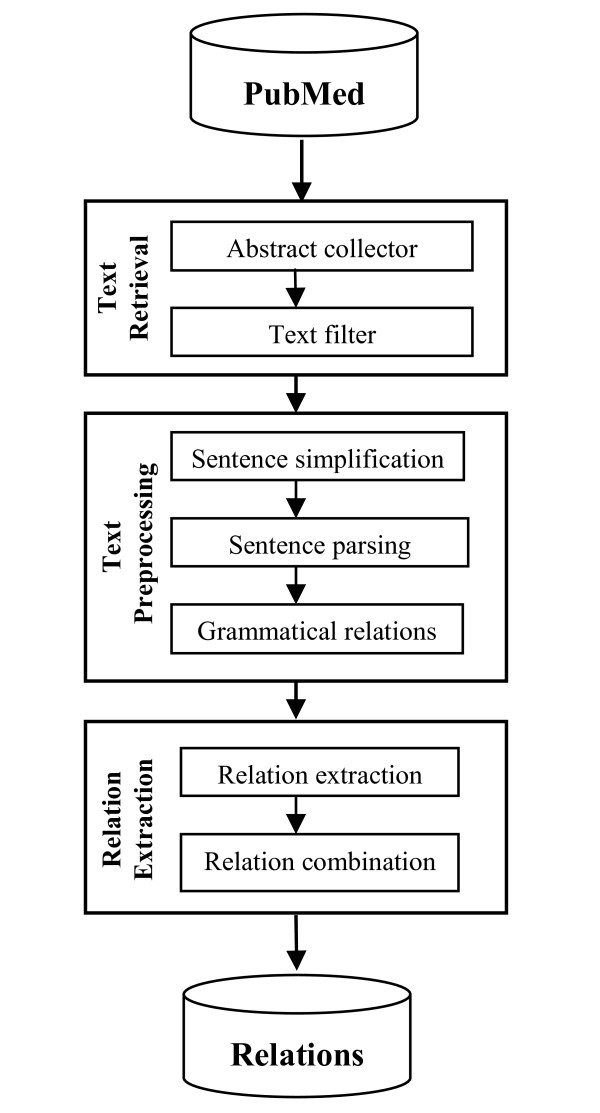
**Work flow of the system**.

The work-flow of the proposed method is shown in Figure 1. The system consists of the following components:

1. Text retrieval

2. Text preprocessing

3. Relation extraction

The text retrieval component collects relevant abstracts from PubMed and filters out irrelevant sentences. The text preprocessing component then simplifies sentences, parses them using the Stanford Lexicalized Parser version 1.6 [26] and applies grammatical relations to generate sentence components. The relation extraction component applies a set of rules to sentence components to extract candidate relations. Finally, the extracted relations are combined using a logistic regression classifier.

As the combination of relation extraction methods has previously been shown to increase overall system performance [23,27], we decided to combine both co-occurrence and NLP methods to enhance the overall performance. For the text retrieval, we apply co-occurrence to obtain high sensitivity, while in the relation extraction phase, we apply NLP and rule-based methods to gain high specificity.

### Text retrieval

The text retrieval phase consists of two steps: collection of abstracts and selection of candidate sentences from those abstracts. To collect relevant abstracts, we prepare a list of drug names by collecting them from websites related to HIV treatment such as the Stanford HIVDB and RegaDB. The system queries PubMed using the drug names as keywords. The obtained abstracts in XML format are parsed using the LingPipe parser http://alias-i.com/lingpipe and then stored in a local database. Next, abstracts are split into sentences and the system selects candidate sentences that belong to either one of the following cases:

• A single sentence: if a sentence contains at least one mutation and one drug then it is selected.

• An inter-sentence: if two sentences are adjacent, one sentence contains at least one drug, and the other contains at least one mutation, then these sentences are selected.

In order to identify mutations in text, the system uses regular expressions. The regular expression for a mutation consists of an optional single letter code for the amino acid followed by a position consisting of one to three digits and ending with an amino-acid code letter [28]. Examples are K65R, I84V, and 103N. Groups of amino acids which can appear as mutations at a single position are notated with the separators "/" or "-", such as 54A/M/V.

### Text preprocessing

The text preprocessing phase consists of three steps: simplification of sentences, parsing the simplified sentences, and generating grammatical relations.

#### Simplifying sentences

Generic English parsers tend to perform poorly when applied directly to biomedical texts [29]. This is because the sentences in abstracts for such texts frequently use long and complex noun phrases and contain technical terms which are specific to the biomedical domain. For these reasons we simplify the sentences in a number of ways to make them more amenable to the parser. This process has been proposed in previous work by [15]. We further enhance this process by grouping mutations and drugs. The simplification process consists of 5 steps:

##### Removing parenthetical remarks

Words inside a pair of parenthesis () are removed except those that contain drug names or mutations.

##### Replacing "known" terms

Common terms such as "human immunodeficiency virus type 1 (HIV-1)" are replaced by their well established abbreviations.

##### Grouping mutation and drug names

The drug names and or mutations in sentences are replaced by a predefined name. In case there is an enumerated list of drug names/mutations (either conjunctive or disjunctive), the system also replaces this group by a new name. For each sentence, the system maintains a list of generated words with the original words as a reference to be used in the extraction phase.

##### Normalizing sentences

Special characters, such as "-", "+" or "/" between words, may cause parse errors and are therefore removed.

##### Anaphora resolution

A simple anaphora resolution algorithm is implemented to resolve a list of predefined pronouns such as *this drug*, *these drugs*, etc., which refer to drug names or mutations in the sentence.

The following example illustrates the result of this simplification process:

• Original sentence: '*A371V and Q509L increased resistance to lamivudine and abacavir, but not stavudine or didanosine'*.

• Simplified sentence: '*MUTATION0 increased resistance to DRUG0, but not DRUG1*'.

##### Recognized keywords

In addition to mutations and drug names, the system also recognizes *relation words *and *manner words*. We prepared a list of relation words that indicate causal relations between drugs and mutations by manually analyzing sample sentences. This list is shown in Table 1. Furthermore, during this process we also collected adjectives and adverbs that describe the '*manner*' of the relation such as *high, strong, full, low, weak*, etc., as shown in Table 2. For each sentence, a list of these keywords is maintained and used in the extraction phase. For more details of the simplification process see Additional file 1.

**Table 1:** Examples of relation words and their categories

Resistant	Susceptible	Associated	Responsive
Resistance, resistant, antagonize	Susceptibility, susceptible, sensitivity	Associate, association, bind, incorporation	Response, responsible

**Table 2:** Examples of manner words and their corresponding groups.

High	Increase	Medium	Decrease	Low	No manner
High, full, strong, significant	Increase, higher	Intermediate, medium, moderate	Decrease, reduce, lower, diminished	Low, weak, loss	

#### Parsing sentences and generating grammatical relations

**Figure 2:**
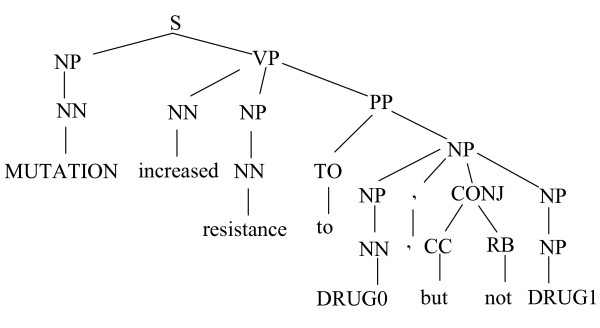
**Penn Treebank output of the Stanford parser**.

Before parsing, each simplified sentence is checked for a triplet <mutation, relation, drug>, in which mutation and drug are predefined names resulting from the simplification process. Sentences containing the required triplet are parsed. The parser generates the output in the form of the Penn Treebank. Figure 2 shows an example of the Stanford parser output. The input for the parser is the simplified sentence of the previous step.

The parse trees are then subjected to a set of English grammatical relation rules which is bundled with the parser to generate sentence constituents such as subject, object, preposition etc, which are then used as the input of relation extraction phase. The built-in rule set consists of 49 rules, however, we only apply 11 rules that generate the most common relations, and this is shown in Table 3.

**Table 3:** Main grammatical relations and some of their values generated from parse tree in Figure 2.

Component	Explanation and example
nsubj	Nominal subject: MUTATION0
nsubjpass	Nominal passive subject
pre	Predicate of a clause: increased resistance to DRUG0, but not DRUG1
dobj	Direct object: resistance
iobj	Indirect object
pobj	Prepositional object: DRUG0, but not DRUG1
prep	Prepositional modifier: to DRUG0, but not DRUG1
cc	Coordination: but not
conj	Conjunction: DRUG1
neg	Negation: not
acomp	Adjectival complement

### Relation extraction

Most of the relations in biomedical texts in the English language can be expressed in two main forms:

• *Clause form*: a relation between entities is expressed by a relational verb in the form of subject and predicate (A - relation - B).

• *Phrase form*: a relation between entities is expressed by a relational noun and makes use of prepositions to connect entities (Relation - A - B).

Based on these relation forms, we define two rules:

***Rule 1a****: *This rule applies to relations in the following form:

This is the most common relation form found in texts. If *keyword1 *is MUTATION then *keyword2 *is DRUG and vice versa. The procedure to extract relation of ***rule 1a ***is carried out as follows:

• *Input*: lists of sorted relation words, manner words, predefined keywords and components that belong to the predicate of the current clause.

• *Requirement*: The nominal subject (nsubj) must contain a predefined keyword (MUTATION/DRUG).

*Step 1*: Find a relation pair:

a. Pick a relation word from the sorted list of relation words.

b. Find a keyword from the sorted list of predefined keywords at distance 1 to 4 words from the relation word. This keyword either belongs to the same component as the relation word or belongs to an adjacent component. If found, go to step 2, otherwise pick another relation word from the list.

*Step 2*: Find manner words:

a. Find a manner word from the sorted list of manner words at distance 1 to 3 words from the relation word, this manner word either belongs to the same component as the relation word or belongs to an adjacent component.

b. Continue to find other manner words at distance 1 to 2 words from this manner word.

*Step 3*: Extract a relation:

a. Form a pattern to extract a relation with the data found in step 1 and step 2.

b. If the list of relation words is not empty then go to step 1.

*Step 4*: Extract relations from a conjunction component (conj) that only contains a predefined keyword:

a. If a relation pair is found adjacent to this conj component, then use the relation word that is closest to the keyword of this component to form a relation pair.

b. Find a manner word in a similar approach as step 2.

c. Form a pattern to extract the relation from this component.

Note: In case there is more than one predefined word in nsubj, the first keyword is selected then the procedure will repeat for the other keywords.

**Rule 1b**: The same as rule 1a, but switch the role of subject and predicate for passive sentences.

**Rule 1c**: Preposition (*keyword1*) + Predicate (*Relation word *+ *key-word2*)

This rule is similar to rule 1a, but instead of looking for a predefined keyword in nsubj, the system finds a predefined keyword in the preposition component (prep) that is located before the predicate.

**Rule 2**: This rule is applied to relations in a phrase form. First, the system calculates distance (measured by word) and numbers of occurrence of each of the following pairs in the current phrase: <Mutation, Relation>, <Drug, Relation>, <Relation, Mutation>, <Relation, Drug>. Based on these values, a heuristic algorithm forms a triple <relation, keyword1, keyword2>. Secondly, searching for *manner words *is done in the same way as described for rule 1a.

#### Check for negation

**Figure 3:**
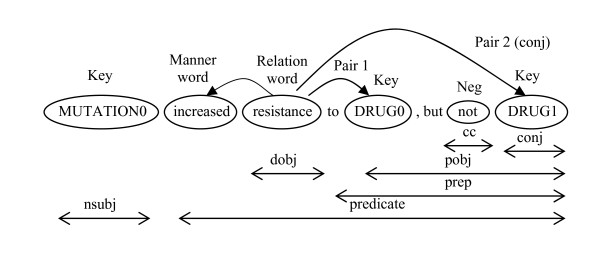
**Extracting relations from grammatical relations of a simplified sentence**.

We can classify the negation into two cases: the negation words located outside and inside a relation. We only focus on the case where negation words are located inside a relation (see Figure 3). The other case is ignored since its frequency is very low and requires extensive semantic analysis. A more comprehensive analysis of negation can be found in [30]. Checking for negation is done the same way as checking for manner words.

Depending on the sentence components generated for each sentence, the system then decides to apply rule 1a, 1b or 1c, when these rules fail to extract relations, rule 2 is applied. For example, when applying rule 1a to the sentence components of the sentence in the previous step, the system forms the relation pairs as shown in Figure 3. The extracted relations are as follows:

MUTATION0 increased resistance to DRUG0

MUTATION0 not increased resistance to DRUG1

#### Post relation extraction

The keywords in the extracted relations are then replaced with the original mutations or drug names. Next this group of drug names and mutations are disentangled and split into single values. For example, with the extracted relation: 'MUTATION0 increased resistance to DRUG0'. The relation after replacing keywords is as follows:

A317V and Q509 L increased resistance to 3TC and ABC.

### Relation combination

The extracted relations obtained in the relation extraction step are expressed in different manners and may also contain contradictory relations. In addition, these relations are usually taken out of context so they do not represent the true nature of the relation as it was specified in original sentences. Our task here is to determine a resistance type for each <mutation, drug> pair from these pieces of evidence, which can have the following properties:

- *Containing false positive relations due to relation extraction method or relations are taken out of context*.

- *Relations are in textual descriptive form with different manners, and come from different sources*.

- *Extracted relations contradict with each others (resistant vs. susceptible), this is the most common case*.

Table 4 shows examples of extracted relations between K65R mutation and D4T. The relation combination process is carried out in two steps: grouping relations with the same mutations and drugs, and calculating the resistance type for each <drug, mutation> pair.

**Table 4:** Output extracted relations between K65R mutation and D4T when running the system over all candidate sentences.

Mutation	Relation	Drug
K65R	resistance to	D4T
K65R	reducing resistance to	D4T
K65R	resistance to	D4T
K65R	resistance to	D4T
K65R	resistance to	D4T
K65R	resistance to	D4T
K65R	result to	D4T
K65R	increased susceptibility to	D4T
K65R	fully susceptible to	D4T
K65R	fully susceptible to	D4T
K65R	resulted in reduced susceptibilities to	D4T

#### Grouping relations

First, the mutations in each relation are checked for consistency. Mutations with amino acid letters and those without amino acid letters are converted to a standardized form. Mutations ending with more than one amino acid letter are split into an atomic mutation, for instance M184I/V is converted into two atomic mutations, M184V and M184I. Second, extracted relations that have the same drug and mutation are put into the same group. In each group, the relations are categorized into 4 subgroups according to their resistance properties: resistant, susceptible, responsive, and associated. In addition, negative relations are removed from each subgroup. The categorizing process uses a list of predefined *relation words *some of which are shown in Table 1.

#### Calculating resistance types

**Figure 4:**
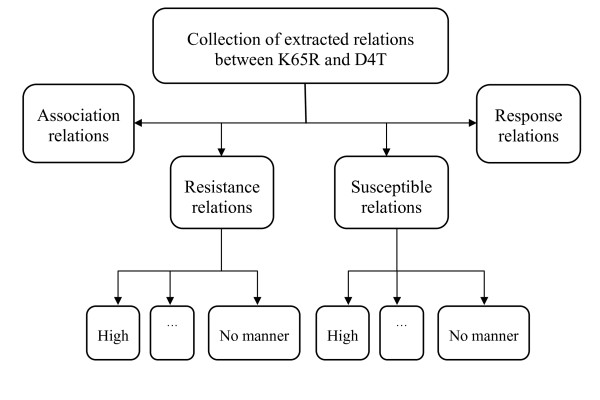
**Example of categorizing extracted relations of the K65R mutation and D4T**.

Since the relations in the association and response subgroups do not indicate clear evidence on drug resistance, we only use relations from the resistance and susceptible subgroups to calculate a resistance value. For each subgroup, we divide relations into six subsets based on their *manner words *that indicate the degree of the relation. Example of the *manner words *is shown in Table 2. The result of this division leads to 12 subsets of relations as illustrated in Figure 4. Since the resistance value for common <drug, mutation> pairs are available in expert systems such as Stanford HIVDB or RegaDB, we transform the current problem into a well-known regression problem [4,31,32] that is to predict the resistance value for each <drug, mutation> pair. We use the output for each <drug, mutation> pair from Stanford HIVDB as the gold standard, and use the number of extracted relations in each subset as feature values. Now the problem is to find optimized weight factor for each subset in the following equation:(1)

where E(y) is the predicted value of the <mutation, drug> pair y; r_i _and s_i _are the number of relations in each subset of the resistance and susceptible subgroup {high, increased, medium, decreased, low, no manner} and w_j _(j = 1, ..., 12) denote the corresponding weights of these sets.

The procedure to determine the weight factors as follows:

- Divide the extracted relations into two datasets, one for determining the weight factors (learning) and one for testing. Extracted relations for each <drug, mutation> pair can belong to either datasets. This is to make sure that learning data and test data do not overlap thus avoiding bias the final results.

- Only select a <drug, mutation> pair for training if it has at least 3 extracted relations. Assign the resistance value (resistance/susceptible) for this pair by taking this value from Stanford HIVDB.

- Use the logistic regression function from WeKa package version 3.6 [33] to find the weigh factors.

When the weight factors in equation 1 are obtained, we then apply these values to predict the resistance value for the remaining data. Again, these predicted values are compared with the output from HIVDB system.

## Results and discussion

### Datasets

We use the text retrieval component with a list of 22 FDA approved drugs (see Additional file 2) to collect candidate abstracts from the PubMed database. We obtained 129,448 unique abstracts when the search was carried out on June 10, 2009. Among these collected abstracts there were 74,321 abstracts containing at least one drug name in the body text, 9,651 abstracts contained mutations, and 5,615 abstracts contained both drugs and mutations. When applying the text filter to find candidate sentences, we obtained 2,937 candidate sentences which contained both drugs and mutations. Of these 1,913 were single sentences and 1,024 were inter-sentences that contained the triple <mutation, relation, drug>.

### Evaluation metrics

We use recall, precision, and the F-score as metrics to evaluate the performance of our system to extract relations based on the following calculations:

where TP, FN, and FP are defined as:

TP (true positives): is the number of relations that were correctly extracted from input sentences.

FN (false negatives): is the number of relations that the system failed to extract from input sentences.

FP (false positives): is the number of relations that were incorrectly extracted from input sentences.

The F-score is the harmonic mean of recall and precision.

We asked independent medical doctors and virologists from the ViroLab project, with a strong background in drug resistance, to assess the extracted relations.

### Relation extraction performance

In order to evaluate the performance of the relation extraction method, we prepared two datasets: one dataset consist of sentences taken from PubMed abstracts and the other consists of sentences taken from the Stanford HIVDB comments which derived from full text papers. Table 5 gives an overview of the number of positive and negative relations in two datasets.

**Table 5:** Datasets statistics

Dataset	Number of instances	
	
	Positive	Negative
500 sentences from PubMed abstracts	1095	921
130 sentences from Stanford HIVDB comments	307	261

#### Evaluation on PubMed dataset

For dataset from PubMed, there were 1543 out of the 2937 candidate sentences containing a triple <mutation, relation word, drug>. From these, we randomly selected 500 sentences, none of which had been used for developing rules. In this dataset, there are 921 instances of negative relations (46%) and 1095 instances of positive relations (54%). The evaluation by experts against the output of these 500 sentences shows that the system can extract 1023 (896 true positives and 127 false positives) instances of relations with a precision, recall, and F-score of 87%, 82%, and 84.5%, respectively (see table 6).

**Table 6:** Performance of the system compared with the baseline method over 2 datasets.

Datasets	Base_C			Rule based		
	
	P	R	F	P	R	F
500 sentences from PubMed abstracts	53.6	100	69.8	**87.4**	**81.8**	**84.5**
130 sentences from Stanford HIVDB comments	54.4	100	70	**97**	**87**	**91**

Evaluation of the performance (%): precision (P), recall (R), and F-score (F) of the proposed method (rule based) and the co-occurrence baseline method (Base_C)

#### Evaluation of the HIVDB comments dataset

In the second evaluation, we wanted to test the performance of the proposed method on both single and inter-sentences. We used all of 130 sentences taken from the Stanford HIVDB comments, of which there are 32 sentences (24.6%) containing no relation and 98 sentences (75.4%), consisting of 56 single sentences and 42 inter-sentences. Among these 98 sentences, there are 261 instances of negative relations and 307 instances of positive relations. The evaluation shows that the system can extract 275 relations (267 true positives and 8 false positives) with a precision, recall, and F-score of 97%, 87%, and 91.7%, respectively. Table 6 shows the evaluation results of the system over these two datasets. For more details of extracted relations, see Additional file 3, Additional file 4, and Additional file 5 respectively.

#### Analysis of the results

The results in Table 6 show that the performance of the system is comparable with existing relation extraction systems for such tasks as protein-protein interaction or protein-gene interaction, of which most do not take into account the degree of the relations [25,34]. Furthermore, there is no gold standard corpus available to evaluate the results of our system, making it hard to compare the proposed method directly with other relation extraction systems. Therefore, we used a co-occurrence method as a baseline to compare with our method. This method (Base_C) predicts a <mutation, drug> pair occurring in the same sentence as a relation. Table 6 shows that our method has a significantly better performance than the baseline method on both datasets with F-scores of 84.5% and 91% compare to 69% and 70% of the Base_C.

The extraction results of the Stanford HIVDB dataset show a higher precision than the extraction results of datasets taken from abstracts. The reason is that the sentences taken from the Stanford HIVDB were composed in a clear, consistent way, and expressed the relations in an explicit form. In addition, the mutation and drug names are also written in a standard format and the sentences are relatively short. As a consequence, the system can archive better results. In contrast, sentences taken from abstracts are long and often have complex structures and thus are prone to more errors.

To identify the source of the errors, we analyzed the sentences that the system failed to extract (false negative) or extracted incorrectly (false positive). The causes of these errors are parser errors, non specific rules, semantic problems, negation, and anaphora resolution:

##### Parser errors and grammatical relation errors

The most frequent errors were caused by the parser (23/62 i.e. 23 of the 62 failures were due to parser errors). Since the parser is not trained on biomedical texts, it often returns inaccurate parse trees, which in turn generate incorrect grammatical relations. As a result, the system applies inappropriate rules to extract relations. For instance, "*G48 M causes high-level SQV resistance and intermediate resistance to NFV, ATV, IDV, and NFV*". In this example, the parser returns a parse tree in the form of noun phrase (NP) instead of a clause form. However, in some cases, the system can still extract relations by applying rule 2 on noun phrase such as this example.

##### Non specific rules

The second major source of errors is due to cases where the rules are not covered (18/62), as for instance in: "*Additional insertion of M184V into the zidovudine background doubled the resistance, whereas 44/118 did not lead to a further increase*". In such cases, the distance from relation word to keyword is longer than the defined values set in the rules. This can be corrected by relaxing the defined rules; however, this would also mean reducing the precision.

##### Semantic problems

In some cases, the errors were caused by semantic problems (12/62). This occurred when a relation is implied or hyponyms are used. For example: "*The PI mutation I50L causes clinically relevant resistance and increased susceptibility to atazanavir and other PIs respectively*".

There were only a very few cases where the source of error was caused by negation or anaphora resolution. Currently we do not take those sparse situations into account.

### Relation combination performance

We extracted relations from all candidate sentences of the collected abstracts and obtained 2,434 extracted relations. After grouping relations and dropping relations belong to response and association groups, we obtained 612 <mutation, drug> pairs. However, among these, there were only 163 pairs containing more than or equal 3 extracted relations. We selected 63 <mutation, drug> pairs for training the logistic regression function. The remaining 100 pairs are used to predict resistance values. To evaluate the results of the relation combination process, we selected relations of the 10 most common mutations. For each <mutation, drug> pair to be chosen as output relations, it is required that this pair has at least 3 extracted relations from the text. In addition, we have also calculated the resistance type based on three levels of resistance: susceptible (S), intermediate resistant (I) and resistant (R) using the same method as proposed for two levels. Table 7 shows examples of the output of our system on K65R mutation and its related drugs.

**Table 7:** Prediction results of mutation K65R and its related drugs.

Mutation	Drug	Resistance type	HIVDB	REGADB
K65R	3TC	I	I	I
K65R	ABC	I	I	S
K65R	AZT	S	S	S
K65R	D4T	I	I	S
K65R	DDI	I	I	I
K65R	FTC	I	I	I
K65R	TDF	I	I	R
K65R	DDC	I	N/A	N/A

The results of K65R mutation and its drug resistance value calculated by the system compared with the result of the Stanford HIVDB based on three levels of resistance: susceptible (S), intermediate resistant (I), and resistant (R). In addition, we also provided the output of the RegaDB to show the differences between the expert systems.

The result in Table 7 shows that the output of our system has the same resistance type compared with the Stanford HIVDB on K65R mutation. There are 3 relations that have a different resistance type between the Stanford HIVDB and the RegaDB. This discordance is due to the fact that there are cases where RegaDB does not take into account the single <mutation, drug> pairs, therefore the RegaDB gives as an output "susceptible", e.g. in case of <K65R, D4T> and <K65R, ABC> pairs. In contract, our system and the Stanford HIVDB do have evidence for these resistance pairs. In addition, there is one relation that only appears in our system i.e. "K65R-intermediate resistance-DDC".

Table 8 shows a summary of the output results of the 10 common mutations, which account for 33% of extracted relations (615 instances over 54 <mutation, drug> pairs) and cover 3 common drug classes (PI, NRTI, NNRTI). The results are compared manually with the Stanford HIVDB system. The percentage of the agreement between two systems based on two levels of resistance (S, R) are 85%, and based on three levels of resistance (S, I, R) are 76%. By following the reference links provided by the Stanford HIVDB, we discovered that the main reason for the differences of the output between our system and the Stanford HIVDB is that there are many relations which can only be found from full texts, not from abstracts. In addition, the Stanford HIVDB also uses experimental data (e.g., n-fold value of resistance), while our system only uses pure text to synthesise the relations.

**Table 8:** Summary of the prediction results of the 10 most frequent mutations and their related drugs extracted from text compares with the HIVDB on two levels and three levels of resistance: susceptible (S), intermediate resistant (I), and resistant (R).

Mutation	Drugs	Agreement with the Stanford HIVDB output (%)	
		
		Two levels: S, R	Three levels: S, I, R
I84V	ATV, IDV, LPV, NFV, SQV, TPV	6/6	6/6
K103N	AZT, DLV, EFV, NVP	3/4	6/6
K65R	3TC, ABC, AZT, D4T, DDI, FTC, TDF	7/7	7/7
L74V	3TC, ABC, AZT, D4T, DDI	3/5	3/5
L90M	ATV, IDV, LPV, NFV, SQV	5/5	4/5
M184V	3TC, ABC, AZT, D4T, DDI, EFV, FTC, NVP, TDF	6/9	7/9
M46I	ATV, IDV, NFV, SQV	3/4	2/4
Q151M	3TC, ABC, AZT, D4T, DDI	5/5	3/5
V82A	IDV, LPV, NFV, SQV	4/4	3/4
Y181C	AZT, D4T, DLV, EFV, NVP	4/5	3/5

**Over all**		**85%**	**76%**

Furthermore, there are many pairs of <mutation, drug> where the number of extracted relations is below the threshold we have set, so these pairs are not considered by the system and do not appear in the output results. However, we also discovered that our system can extract new relations that do not appear in the Stanford HIVDB as shown in example of K65R mutation above.

#### Atomic value vs. group values

The atomic relations obtained by splitting a group of mutations from original relations are also the cause for disagreement between the output of the system and the Stanford HIVDB result. This was due to the fact that, in some contexts, the resistance only occurs if these mutations come together, but does not occur in a single mutation. For future work, we will take this issue into account.

PubMed abstracts are certainly a good source for extracting causal relations on HIV drug resistance; however, the number of extracted relations from abstracts is relatively low, only 5% of abstracts may potentially provide evidence for drug resistance. Therefore a more advanced form of publishing as proposed in [35] might provide a better solution for collecting data. In addition processing of full texts can be considered. The system can be used as annotator to extract relations from full text articles, the results are then considered as raw relations, which can be evaluated by experts. This system can save experts a significant amount of time otherwise spent finding relevant sentences which provide evidence for drug resistance. For convenience, the system provides summarized data and original texts, from which the relations were extracted, to support the experts in the verification of the results.

## Conclusions

We have proposed a new method for extracting causal relations between drugs and mutations by applying two rule sets over grammatical relations. Our system can extract relations from single sentences and from inter-sentences. In addition, by grouping mutations and drugs, the system also reduces the number of conjunctions and the enumeration lists of entities, thus making the process of extracting relations much quicker and less error prone.

We have also described a method to combine extracted relations which combines the manner of each individual relation and deals with contradictory relations in order to determine resistance type for each <drug, mutation> pair. The output of the relation combination shows promising results with 85% and 76% agreement to the Stanford HIVDB on two and three levels of resistance respectively. Furthermore, the system can also provide new relations and additional sources of evidence to analyze the discordance between expert systems.

The proposed algorithm uses publicly available NLP tools. Therefore, it is easy to setup a similar system, and it is suitable for extracting relations in case where an annotated corpus is not available. The algorithm can also be applied to extract other types of relations in which entities have a distinct category such as gene-protein, gene-disease, and disease-mutation. In such cases, the system needs to provide a list of relation words, manner words, and Name Entity Recognition (NER) module.

The performance of the system is adequate. The system processed 129,448 abstracts on a Centrino Duo 1.8 GHz laptop in 35 minutes, of which 97% of the time was used by the parser, 2% was used for filtering and simplification of the sentences, and 1% of the time was used for the actual extraction and combination of the relations. The system is clearly capable to be used in large-scale relation extraction experiments. The system is used in 5 hospitals from the Virolab project http://www.virolab.org to preselect the most relevant novel resistance data from literature and present those to virologists and medical doctors for further evaluation.

## Authors' contributions

The results reported in this paper are part of the PhD research of the first author, QCB. BON conceived the study and made the initial design. PMAS, BON and CAB participated in the analysis, editing the paper and as PhD supervisors. All authors read and approved the final document.

## Supplementary Material

Additional file 1 **Simplification_process**. A MS Word document provides details of simplification process. Click here for file

Additional file 2 **List_of _approved_HIV_drugs**. A MS Word document provides a list of 22 FDA approved drug names. Click here for file

Additional file 3 **500_PubMed_results**. A text file containing the list of 500 sentences taken from abstracts and the extracted relations corresponding to each input sentence. Click here for file

Additional file 4 **130_HIVDB_results**. A text file containing the list of 130 sentences taken from the Stanford HIVDB rules and the extracted relations corresponding to each input sentence. Click here for file

Additional file 5 **Performance_evaluation**. A MS Word document provides details of the evaluation of the extraction method on 500 sentences taken from PubMed abstracts. Click here for file
