# Supplementary material for: Extracting causal relations on HIV drug resistance from literature
Source: BMC Bioinformatics. 2010 Feb 23;11:101. doi: 10.1186/1471-2105-11-101 (PMC2841207; doi:10.1186/1471-2105-11-101)
Supplement: Additional file 1 — Simplification_process. A MS Word document provides details of simplification process. [file 1471-2105-11-101-S1.DOC]

**DETAILS OF THE SIMPLIFICATION PROCESS**

The simplification process is carried out in 5 steps:

**1.** *Removing parenthetical remarks* Words inside a pair of parenthesis () are removed unless the parenthesis contains drug names or mutations.

**2.** *Replacing "known" terms*  Common terms such as "human immunodeficiency virus type 1 (HIV-1)" are replaced by their well established abbreviations by using a list of predefined terms. Some examples of predefined terms are shown in the following table:

| **If *occurred* in text** | **Then *replaced* with** |
| --- | --- |
| tenofovir disoproxil fumarate | TDF |
| Tenofovir DF | TDF |
| 2',3'-dideoxy-3'-thiacytidine | 3TC |
| 2',3'-dideoxy-5-fluoro-3'-thiacytidine | FTC |
| reverse transcriptase (RT) | RT |
| nucleoside reverse transcriptase inhibitors (NRTI) | NRTI |
| human immunodeficiency virus type 1 (HIV-1) | HIV1 |
| thymidine analogue mutations (TAMs) | TAMs |

**3.** *Grouping mutation and drug names* The drug names and/or mutations in sentences are replaced by a predefined name. In case there is an enumerated list of drug names/mutations (either conjunctive or disjunctive), the system also replace this group with a new name. For each sentence, the system maintains a list of generated words with the original words as a reference to be used in the extraction phase. The patterns (Java regular expression) for grouping mutation and drug names as follows:

1. *Single mutation pattern*:

m_pattern = \b(?<!\.)([ACDEFGHIKLMNPQSTWVY])?(\d){2,3}+[ACDEFGHIKLMNPQSTWVY](/[ ACDEFGHIKLMNPQSTWVY])*\b

1. *Group mutations pattern*:

g_pattern = m_pattern ((\s)?(/|\+)?(,)?(\s)?(and|or)?(\s)?m_pattern)*

1. Procedure for grouping drug names:

**Step 1**: Replace drug names (see supplementary file for drug-names) in text with the predefined words: DRUG+x ; x start from 0.

**Step 2**: Apply the following pattern to group an enumerated list of drug

g_drug = DRUG\d+((\s)?(/|\+)?(,)?(\s)?(and|or)?(\s)?DRUG\d+)*

**4.** *Normalizing sentences* Special characters, such as "-", "+" or "/" between words, may cause parse errors and are therefore removed. Table below shows some examples of patterns that are used to for normalizing sentences.

| Pattern (regular expression) | Replaced with |
| --- | --- |
| (?=\d)- | Blank |
| -|+|/|\ | Blank |
| \s{2,} | Blank |

**5*.*** *Anaphora resolution* A simple anaphora resolution algorithm is implemented to resolve a list of predefined pronouns such as *this drug*, *these drugs*, etc., which refer to drug names or mutations in the sentence. Table below shows a list of predefine pronouns used to resolve mutation/drug names.

| **Pronoun** | **Rule/constraint to resolve the reference pronoun** |
| --- | --- |
| This drug | If these pronouns occur in the text, then check nominal subject for drugs mentioned that have the same quantity (the number of drugs). If not found, then find the closest drugs mentioned that have the same quantities. Replace the pronoun with the value found. |
| These drugs |
| Both drugs |
| Two drugs |
| Three drugs |
| This mutation | If these pronouns occur in the text, then check nominal subject for mutations mentioned that have the same quantity (the number of mutations). If not found, then find the closest mutations mentioned that have the same quantities. Replace the pronoun with the value found. |
| These mutation |
| Two mutations |
| Both mutations |
| Three mutations |

**Performance evaluation of the simplification process:**

In order to evaluate the performance of the simplification process, we randomly select 500 sentences out of the 2937 candidate sentences, each of which contains at least a drug name and a mutation. Among these there are 238 sentences (47.6%) containing no relation and 262 sentences (52.4%) containing relations between drugs and mutations. After the simplification process, the system filtered out 244 sentences, of which 238 sentences are true negatives and 6 sentences are false negatives with a precision, recall, and F-score of 97.5%, 100%, and 98.7%, respectively.
