# Supplementary material for: Extracting causal relations on HIV drug resistance from literature
Source: BMC Bioinformatics. 2010 Feb 23;11:101. doi: 10.1186/1471-2105-11-101 (PMC2841207; doi:10.1186/1471-2105-11-101)
Supplement: Additional file 2 — List_of _approved_HIV_drugs. A MS Word document provides a list of 22 FDA approved drug names. [file 1471-2105-11-101-S2.DOC]

**LIST OF FDA APPROVED HIV DRUG NAMES**

A. **Nucleoside/Nucleotide Reverse Transcriptase Inhibitors (NRTIs)**:

| Abbreviation | Generic name | Brand name |
| --- | --- | --- |
| 3TC | Lamivudine | Epivir |
| ABC | Abacavir | Ziagen |
| AZT or ZDV | Zidovudine | Retrovir |
| d4T | Stavudine | Zerit |
| ddI | Didanosine | Videx EC |
| FTC | Emtricitabine | Emtriva |
| TDF | Tenofovir | Viread |

B. **Non-Nucleoside Reverse Transcriptase Inhibitors (NNRTIs)**:

| Abbreviation | Generic name | Brand name |
| --- | --- | --- |
| DLV | Delavirdine | Rescriptor |
| EFV | Efavirenz | Sustiva (US) |
|  |  | Stocrin (Europe) |
| ETR | Etravirine | Intelence |
| NVP | Nevirapine | Viramune |

C. **Protease Inhibitors (PIs):**

| Abbreviation | Generic name | Brand name |
| --- | --- | --- |
| APV | Amprenavir | Agenerase |
| ATV | Atazanavir | Reyataz |
| DRV | Darunavir | Prezista |
| IDV | Indinavir | Crixivan |
| NFV | Nelfinavir | Viracept |
| RTV | Ritonavir | Norvir |
| SQV | Saquinavir | Invirase |
| TPV | Tipranavir | Aptivus |

**D. Fusion or Entry Inhibitors:**

| Abbreviation | Generic name | Brand Name |
| --- | --- | --- |
| T-20 | Enfuvirtide | Fuzeon |
| MVC | Maraviroc | Celsentri (Europe) |
|  |  | Selzentry (US) |

**E. Integrase Inhibitors:**

| Abbreviation | Generic name | Brand Name |
| --- | --- | --- |
| RAL | Raltegravir | Isentress |
