# Supplementary material for: Extracting causal relations on HIV drug resistance from literature
Source: BMC Bioinformatics. 2010 Feb 23;11:101. doi: 10.1186/1471-2105-11-101 (PMC2841207; doi:10.1186/1471-2105-11-101)
Supplement: Additional file 5 — Performance_evaluation. A MS Word document provides details of the evaluation of the extraction method on 500 sentences taken from PubMed abstracts. [file 1471-2105-11-101-S5.DOC]

**PERFORMANCE EVALUATION FOR PUBMED DATASET**

Performance evaluation on 4 types of relations over 500 input sentences, of which each contains at least a triple <mutation, relation word, drug>.

| **Relation type** | **True positives** | **False positives** | **False negative** | **Precision (%)** | **Recall (%)** | **F-Score (%)** |
| --- | --- | --- | --- | --- | --- | --- |
| Resistance | 489 | 54 | 102 | 90.1% | 82.7% | 86.2% |
| Susceptible | 223 | 19 | 53 | 92.1% | 80.8% | 86.1% |
| Response | 64 | 30 | 22 | 68.1% | 74.4% | 71.1% |
| Association | 102 | 24 | 18 | 81.0% | 85.0% | 82.9% |
|  |  |  |  |  |  |  |
| **Over all** | **878** | **127** | **195** | **87.4%** | **81.8%** | **84.5%** |
